# Differential microRNA expression, microRNA arm switching, and microRNA:long noncoding RNA interaction in response to salinity stress in soybean

**DOI:** 10.1186/s12864-022-08308-y

**Published:** 2022-01-20

**Authors:** Chade Li, Wenyan Nong, Shancen Zhao, Xiao Lin, Yichun Xie, Ming-Yan Cheung, Zhixia Xiao, Annette Y. P. Wong, Ting Fung Chan, Jerome H. L. Hui, Hon-Ming Lam

**Affiliations:** 1 Center for Soybean Research of the State Key Laboratory of Agrobiotechnology and School of Life Sciences, The Chinese University of Hong Kong, Hong Kong Special Administrative Region, P.R. China; 2 Simon F.S. Li Marine Science Laboratory, School of Life Sciences, The Chinese University of Hong Kong, Hong Kong Special Administrative Region, P.R. China; 3 BGI Institute of Applied Agriculture, BGI-Shenzhen, Shenzhen, 518120 P.R. China

**Keywords:** microRNA (miRNA), Long noncoding RNA (lncRNA), Soybean, microRNA arm switching, miRNA:lncRNA interactions, Salt stress

## Abstract

**Background:**

Soybean is a major legume crop with high nutritional and environmental values suitable for sustainable agriculture. Noncoding RNAs (ncRNAs), including microRNAs (miRNAs) and long noncoding RNAs (lncRNAs), are important regulators of gene functions in eukaryotes. However, the interactions between these two types of ncRNAs in the context of plant physiology, especially in response to salinity stress, are poorly understood.

**Results:**

Here, we challenged a cultivated soybean accession (C08) and a wild one (W05) with salt treatment and obtained their small RNA transcriptomes at six time points from both root and leaf tissues. In addition to thoroughly analyzing the differentially expressed miRNAs, we also documented the first case of miRNA arm-switching (miR166m), the swapping of dominant miRNA arm expression, in soybean in different tissues. Two arms of miR166m target different genes related to salinity stress (chloroplastic beta-amylase 1 targeted by miR166m-5p and calcium-dependent protein kinase 1 targeted by miR166m-3p), suggesting arm-switching of miR166m play roles in soybean in response to salinity stress. Furthermore, two pairs of miRNA:lncRNA interacting partners (miR166i-5p and lncRNA Gmax_MSTRG.35921.1; and miR394a-3p and lncRNA Gmax_MSTRG.18616.1) were also discovered in reaction to salinity stress.

**Conclusions:**

This study demonstrates how ncRNA involves in salinity stress responses in soybean by miRNA arm switching and miRNA:lncRNA interactions. The behaviors of ncRNAs revealed in this study will shed new light on molecular regulatory mechanisms of stress responses in plants, and hence provide potential new strategies for crop improvement.

**Supplementary Information:**

The online version contains supplementary material available at 10.1186/s12864-022-08308-y.

## Background

Soybean is an economically and environmentally important crop, providing a major source of dietary protein and oil for human food and animal feed. Cultivated soybean was domesticated from its wild relatives approximately 5000 years ago in China before being introduced to other parts of Asia 2000 years ago [1, 2]. While Asian countries such as China are major consumers of soybean, it has now become an important cash crop in North and South America for export. Unfortunately, soybean is generally sensitive to saline soil, (often a result of long-term cultivation and over-fertilization of the land), resulting in the reduction of its yield [3]. Earlier studies on salt tolerance of soybean have mainly focused on identifying the protein-coding genes and related mechanisms involved [4].

Besides protein-coding genes, noncoding RNAs (ncRNAs) are equally important regulators of gene expressions in plants, ranging from moderating development to coping with abiotic and biotic stresses. One type of ncRNAs, microRNAs (miRNAs) are typically 21-23 nucleotides long and can bind to the 3′ untranslated regions (3’UTRs) of messenger RNAs (mRNAs), resulting in the downregulation of their target genes. Soybean mutants lacking the miRNA biogenesis component, *Dicer-like 1*, have reduced seed size and defective seedling development [5]. Many other miRNAs were also found to be associated with the development of soybean seeds [6–8], roots [9], and flowers [10]. For instance, the overexpression of miR156b negatively regulated *squamosa promoter-binding protein-like* genes (*GmSPLs*) and postponed flowering time [11, 12]. In addition to the developmental roles, miRNAs are also associated with the responses toward abiotic stresses in soybean [3, 13–17], such as the involvements of gma-miR169c and gma-miR394a in drought tolerance and salt sensitivity [18–20], and miR399a and miR172c in root development under salinity stress [21, 22].

**Fig. 1:**
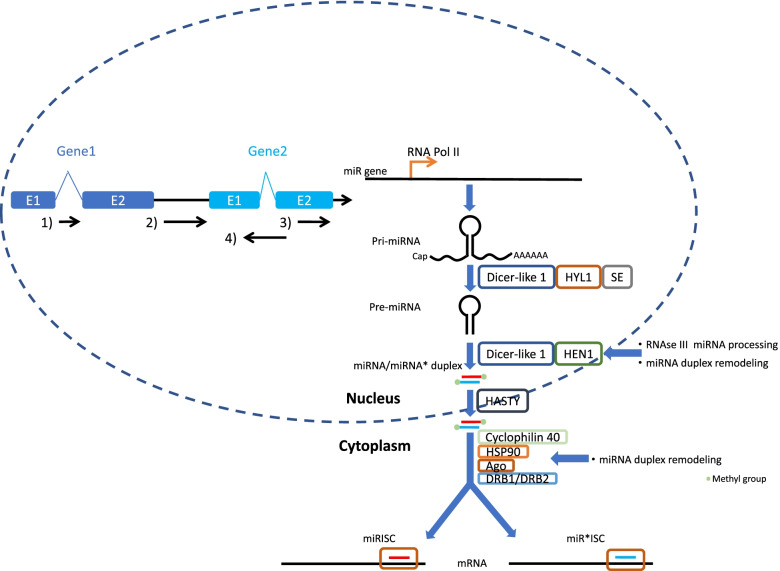
Schematic diagram showing the biosynthesis of microRNAs (miRNAs) and long noncoding RNA (lncRNAs) (modified from [25–27]). miRISC: miRNA-induced silencing complex; miR*ISC: miRNA*-induced silencing complex. E1, E2: exon 1, exon 2; 1) intronic long noncoding RNA, 2) intergenic long noncoding RNA, 3) exonic long noncoding RNA, 4) antisense long noncoding RNA

Despite a better understanding of the different roles of miRNAs in plants including soybean, several aspects remain poorly known, one of which is the phenomenon of miRNA arm-switching ([23, 24]; Fig. 1). In the biogenesis of miRNA, the dominant strand could change depending on cellular contents. Since the complementary strands of mature miRNAs will target different sets of mRNAs, alternative strand selection has been implicated in various biological mechanisms in animals (e.g. [26, 28, 29]). In plants, there are only a handful of studies related to miRNA arm-switching. For instance, miR393 and miR399 could undergo arm-switching in *Arabidopsis* under pathogen infection and phosphate deprivation [30, 31]. The analyses of 29 rice small RNA libraries also revealed miRNA arm-switching in different tissues and under abiotic stresses [32]. Nevertheless, whether miRNA arm-switching could potentially contribute to soybean abiotic stress responses remains unexplored.

Another aspect that requires further investigation is the interactions between miRNAs and other ncRNAs, such as long noncoding RNAs (lncRNAs). Various roles of lncRNAs in gene regulation have already been demonstrated in plants, including transcriptional regulation [33–36] and alternative splicing [37]. LncRNAs could also augment the repertoires of RNA molecules [27]. For instance, the lncRNA IPS1 could interact with the miRNA miR399 to play a role in phosphate homeostasis by regulating its target gene *PHO2* in *Arabidopsis* [38, 39] and *Medicago* [40]; and the lncRNA osa-eTM160 could interact with osa-miRNA160 to regulate rice development [41]. In soybean, there has only been a genome-wide prediction of lncRNAs and miRNAs [42] but no systematic study on their interactions has been carried out yet. Therefore, the interactions between miRNAs and lncRNAs in relation to physiological responses toward abiotic stresses in soybean have remained elusive.

Here, we generated miRNA sequencing data from soybean leaf and root tissues from two soybean accessions (a cultivated *Gylcine max* C08 and a wild *Glycine soja* W05 accession as representatives) treated with NaCl at six time points with three biological replicates, in order to shed light on (i) differential miRNA expression, (ii) miRNA arm-switching, (iii) interactions between miRNAs and lncRNAs; and (iv) conserved and accession-specific responses between cultivated and wild accessions in response to salinity stress in soybean.

## Results

### Differential miRNA expression in soybean under salinity stress

**Fig. 2:**
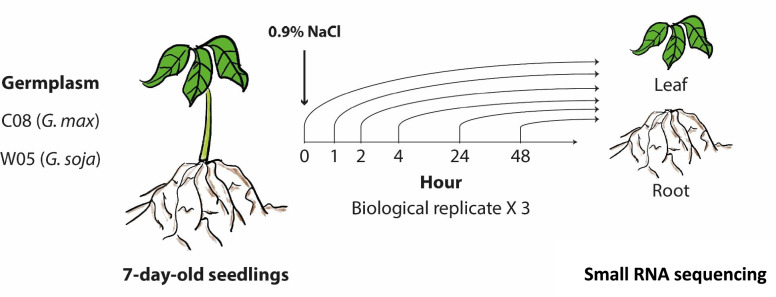
Schematic diagram showing the experimental setup

**Fig. 3:**
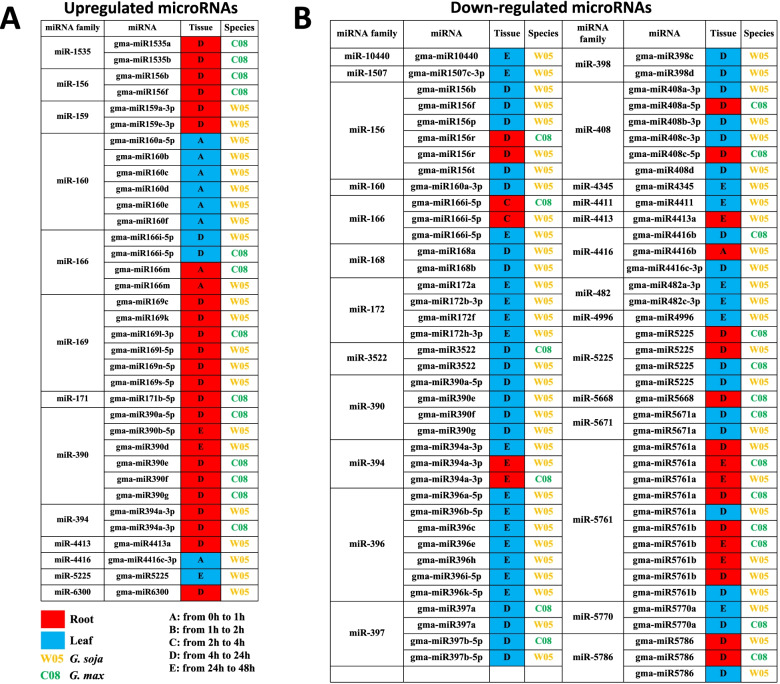
Differential expression of soybean microRNAs (miRNAs) under salinity stress. **A** Up-regulated miRNAs. **B** Down-regulated miRNAs

The setting of the experiment is described in materials and methods and a total of 72 small RNA datasets were generated for analysis (Fig. 2). Details of the datasets are listed in Supplementary Table 2. The small RNA sequencing data generated above were then used to identify miRNA candidates responsive to salinity stress. By comparing samples across different treatments, 35 upregulation and 77 downregulation events were found in the leaf and root between the different time points (Fig. 3).

Among the upregulated miRNA candidates (Fig. 3A), certain members within the same miRNA family sub-functionalized and were differentially expressed at different salinity conditions, including miR166 and miR390. In the case of the miR166 family, the expression of miR166m was found to be upregulated in the root of both W05 and C08 from 0 to 1 h upon salinity treatment, while the expression of miR166i-5p was upregulated in the leaf of both W05 and C08 from 4 h to 24 h. Similar sub-functionalization of expression patterns was also observed in the downregulated miRNAs, for instance, the miR156 family (Fig. 3B).

In addition, divergent expression trends of miRNAs upon salinity stress were also revealed within or between the tissues of the cultivated and wild soybean, including miR156b, miR156f, miR160a, miR166i, miR390a, miR390e, miR390f, miR390g, miR394a, miR4413a, miR4416c, and miR5225 (Fig. 3). For example, the expression of miR156b was upregulated in the root of C08 from 4 h to 24 h, while its expression was downregulated in the leaf of W05 from 4 h to 24 h.

On the other hand, some other miRNAs demonstrated consistent expression responses under salinity stress in the same tissue of both accessions, including miR156r, miR166i, miR3522, miR394a, miR397a, miR397b, miR5225, miR5671a, miR5761a, miR5761b, miR5770a, and miR5786 (Fig. 3). For example, miR156r was downregulated from 4 h to 24 h in the root of both C08 and W05. These miRNAs could play potentially important roles in the response to salinity stress and therefore deserve further investigations (Fig. 3).

### MiRNA arm expression and switching of dominance

In addition to the conventional comparisons of differential expressions in miRNA family members upon salinity stress, we also investigated the expression patterns between the two arms (5p and 3p) generated from the same miRNA locus. Conserved expression patterns between miRNA arms were observed in both soybean accessions, an example of which is miR169l, where both the 5p and 3p arms had increased expression under salinity treatment between 4 to 24 h in the root samples (Fig. 3A).

**Fig. 4:**
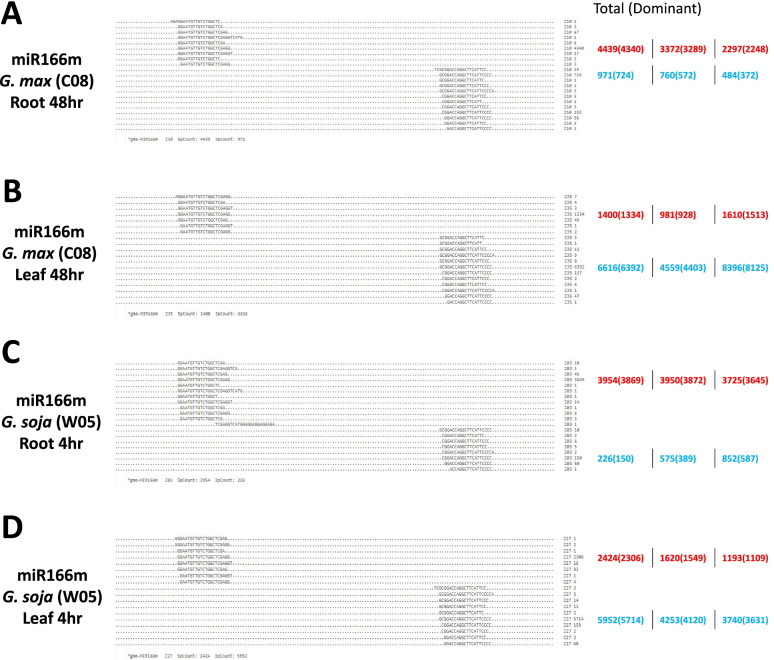
MicroRNA (miRNA) arm-switching of miR166m. **A**, **B** Root (**A**) and leaf (**B**) samples of *G. max* (C08) at 48 h post-salinity treatment. **C**, **D** Root (**C**) and leaf (**D**) samples of *G. soja* (W05) at 4 h post-salinity treatment. Red and blue represent the 5p and 3p arms of miR166m, respectively

However, divergent expression patterns between the two miRNA arms were also observed in the different tissues of the cultivated and wild soybean. For instance, decreased expression of the 3p arm was revealed in miR408a in the leaf tissues of W05 between 4 to 24 h, while downregulation of the 5p arm was observed between 4 to 24 h in the root tissues of C08 (Fig. 3B). Another example is miR408c which represents the same expression pattern as miR408a (Fig. 3B). Next, we explored whether miRNA arm-switching has occurred, using the stringent criteria as described in Materials and Methods, and found that miR166m underwent arm-switching in both soybean accessions (Fig. 4). Specifically, in all three biological replicates of the C08 root samples challenged with NaCl for 48 h, 5p arm dominance was recorded for miR166m (Fig. 4A; Supplementary Fig. 1), and yet at the same time point in the C08 leaf tissue, all three biological replicates exhibited 3p arm dominance for the same miRNA (Fig. 4B; Supplementary Fig. 1). The same pattern was also observed in W05 at 4 h upon salinity treatment, where miR166m exhibited different arm dominance in the root versus the leaf (Fig. 4C & D; Supplementary Fig. 1). In silico prediction of target genes of miR166m-5p and miR166m-3p in C08 and W05 were carried out and summarised in Supplementary Table 5. Conserved targets of the two arms were identified, such as the chloroplastic beta-amylase 1 targeted by miR166m-5p and calcium-dependent protein kinase 1 targeted by miR166m-3p. Predicted target genes regulated by miR-166 m and were previously reported relating to salinity stress were further selected for validation by dual luciferase reporter assay (Supplementary Fig. 3), suggesting arm-switching of miR166m play roles in soybean in response to salinity stress. In any case, this is the first time a robust and confident case of miRNA arm-switching was demonstrated in both cultivated and wild soybean accessions.

### MiRNA and lncRNA interactions in soybean under salinity conditions

**Fig. 5:**
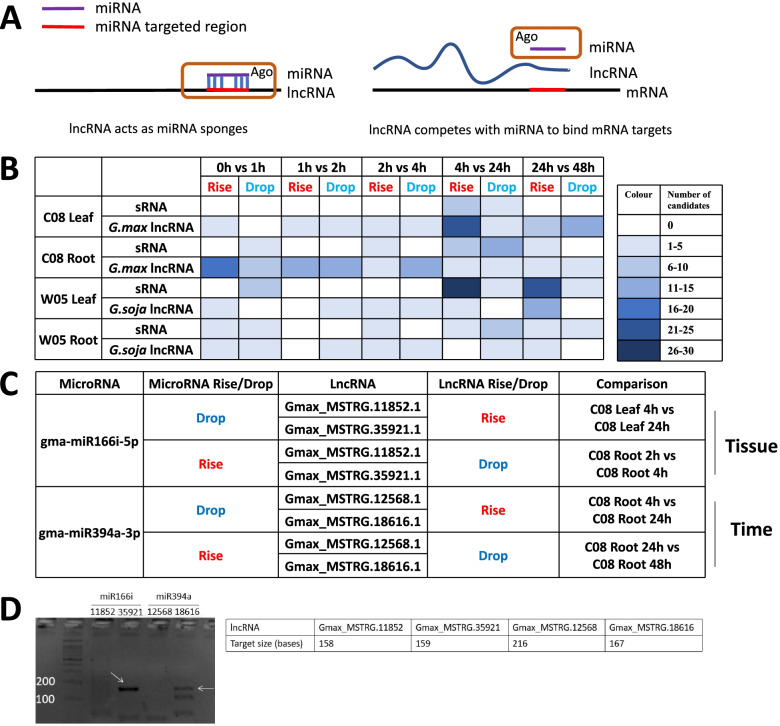
MiRNA:lncRNA interactions in response to salt stress. **A** Schematic diagram showing miRNA:lncRNA interactions (modified from [46, 47]). **B** Numbers of predicted interacting miRNAs and lncRNAs with antagonistic expression patterns. **C** Four predicted cases of miRNA:lncRNA interactions. **D** LAMP assay and RT-PCR validation of miRNA:lncRNA interactions. Arrows indicate the expected sizes of the target lncRNAs

The potential interaction between miRNAs and lncRNAs was then investigated by combining the data generated in this study with our previous studies on soybean transcriptome reprograming in response to salinity stress [43–45]; Supplementary Table 4). Two criteria were used for predicting interactive pairs of miRNA and lncRNA, including: (1) sequence complementarity between the miRNA and the lncRNA, and (2) opposite trends in the expression patterns between the miRNA and the lncRNA upon salinity stress (Fig. 5A & B). Predicted miRNA:lncRNA pairs meeting these two criteria could suggest their functional relevance in salinity stress responses, and a total of four possible miRNA:lncRNA pairs were identified, with miR166i and miR394a each targeting two lncRNAs (Fig. 5C).

Specifically, in C08, miR166i-5p decreased in expression in leaf between 4 and 24 h upon salt treatment, while its predicted target lncRNAs, Gmax_MSTRG.11852.1 and Gmax_MSTRG.35921.1, increased in expression levels (Fig. 5C). On the other hand, in the root, upon salinity treatment between 2 and 4 h, miR166i-5p increased in expression level while the expressions of Gmax_MSTRG.11852.1 and Gmax_MSTRG.35921.1 decreased (Fig. 5C). Using the LAMP assay followed by RT-PCR, the interactions between miR166i and lncRNA Gmax_MSTRG.35921.1 was validated (Fig. 5D). This miRNA:lncRNA pair showed tissue-specific variations in their antagonistic expression patterns during soybean salinity stress.

On the other hand, time-specific variations in expression patterns were observed for miR394a and its predicted target lncRNAs in the root samples of C08, where different antagonistic expression patterns were observed between miR394a and its two predicted targets, Gmax_MSTRG.12568.1 and Gmax_MSTRG.18616.1, at different time points after salt treatment (Fig. 5C). However, only the interaction between miR394a and Gmax_MSTRG.18616.1 could be validated by the LAMP assay and RT-PCR (Fig. 5D). Furthermore, utilising the dual luciferase reporter assay, we validated the potential interactions of these two microRNAs with lncRNAs and genes related to salinity stress, suggesting the potential regulation of these two miRNAs under salinity stress (Supplementary Table 6 and Supplementary Fig. 3).

## Discussion

Previous studies have explored and established the potential roles of soybean miRNAs under salt treatment, ranging from elucidating individual miRNA functions [19, 21] to the genome-wide profiling of miRNAs via transcriptome sequencing [13, 48, 49]. This study further advances the knowledge of miRNAs in several aspects.

In terms of miRNA sequencing data, previous studies have usually focused on a single tissue/time point/accession. For instance, information was provided on a salt-sensitive soybean inbred line “HJ-1” based on a single tissue type at a single time point, i.e. root at 48 h [13]; effects of salt treatment on the leaf and root samples of “William 82” at various early time points (0, 1, 3, 6, 9 and 12 h; [48]); and longer-term effects on the root tissues of “William 82” at 15d after salt treatment [49]. On the other hand, the data generated in this study covers two tissue types (leaf and root) at a large range of time points (0, 1, 2, 4, 24 and 48 h) from two accessions (*G. soja* “W05” and *G. max* “C08”), providing an unprecedented opportunity to understand the various dynamic contributions of miRNAs to soybean physiology in coping with salinity.

In addition to providing a list of differentially expressed miRNAs, this study also uncovered new aspects of miRNA responses to abiotic stresses in plants. Similar to other studies, we found that in this study, members of the same miRNA family exhibited different expression patterns upon salinity challenge, but because we could compare the data between two accessions in the same tissues and time points, we were then able to reveal the conserved and divergent trends of expression patterns of miRNAs, which in turn allowed us to narrow down those miRNAs which are potentially being negatively selected in the salinity responses in soybean, which can then lead us to their target genes that could be the important players in these responses.

This study has also shed new light on how the different arms of the miRNA could contribute to plant physiology. Here we have identified conserved and divergent expression patterns between the two arms across the two accessions. Specifically, the two arms of miR169l exhibited the same expression profiles/response upon salinity challenge. This could potentially be used as another strategy to identify miRNAs which could play important roles in soybean physiology, by screening for those miRNAs with similar expression profiles under abiotic stresses.

**Fig. 6:**
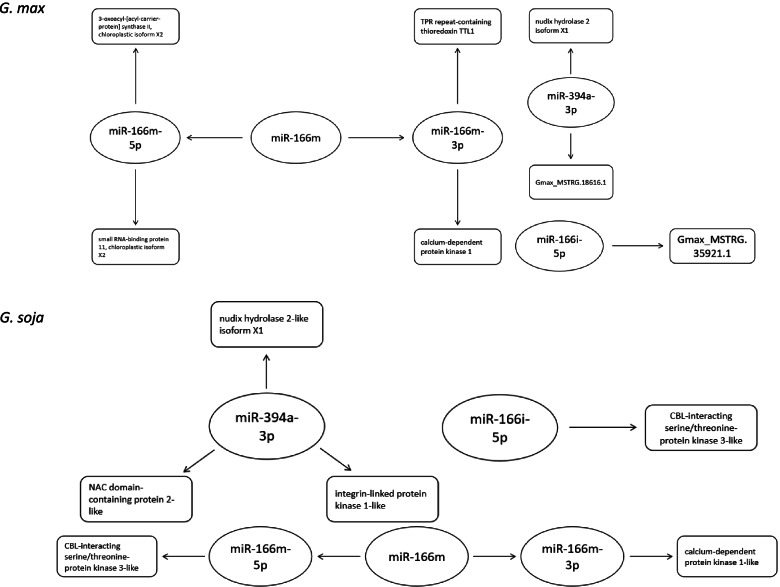
Schematic diagram showing the interactions of miRNA:lncRNA:protein coding genes identified in this study

Furthermore, in animal models, miRNA arm-switching is now well-known as a mechanism for controlling various biological processes, but there are still only limited studies on *Arabidopsis* and rice to understand how such miRNA arm selection could be a potential stress response strategy in plants [30–32]. This study also provided the first documented case of a miRNA (miR166m) undergoing arm-switching in different tissues in soybean, in both *G. max* and *G. soja*, when under salinity stress. Last but not least, we have also revealed for the first time miRNA and lncRNA interactions in soybean under salinity stress (Fig. 6). Previous studies in other plant species have revealed potential miRNA:lncRNA interactions under different conditions, such as phosphate starvation, pathogen infection, and heat stress [47, 50, 51]. Two pairs of miRNA:lncRNA interacting partners (miR166i-5p and lncRNAs Gmax_MSTRG.35921.1; and miR394a-3p and lncRNA Gmax_MSTRG.18616.1) could be identified and validated in soybean under salinity stress. Intriguingly, the interactions between miR166i-5p and its lncRNA interacting partner showed tissue-specific expression patterns, whereas that between miR394a and its lncRNA partner exhibited time-dependent patterns after salt treatment. It has previously been proposed that down-regulated miRNAs may target genes involved in stress responses, while upregulated miRNAs probably target genes involved in development [52]. Validation of the predicted targets for these selected miRNAs indicates the potential regulation of these miRNAs in response to salinity stress given these targets such as SOS2-like protein kinase [53], calcium-dependent protein kinase 1 [54], nudix hydrolase 2 [55] were previously shown to be related to salinity stress. Nonetheless, whether and how these different kinds of noncoding RNAs interaction under salinity stress spatially/temporally, and whether these interactions could contribute to the improvement on salinity tolerance of soybean, remain to be tested.

## Conclusions

This study profiled the time-series differential expression patterns of a group of miRNAs in the root and leaf of two soybean accessions under salinity stress. We also discovered the first evidence of miRNA arm-switching in soybean, indicating that different tissues may have different miRNA arm preference that contributes to different biological functions. Two validated pairs of miRNA:lncRNA interacting partners involved in the response to salinity treatment in soybean were also identified, setting up the foundation for further investigations into the roles of different types of non-coding RNAs in plant physiology.

## Methods

### Soybean treatments and sample collection

Cultivated (C08) and wild (W05) soybean plants were grown and harvested as described in one of our previous publications [43]. Salt treatment was carried out by transferring to half-strength Hoagland solution supplemented with 0.9% (w/v; ~ 150 mM) NaCl when the primary leaves were fully opened. Three independent sets of primary leaf and root samples of each cultivar and treatment were harvested at 0, 1, 2, 4, 24, and 48 h post-treatment. A total of 72 samples were generated.

### Small RNA sequencing

MicroRNA (miRNA) was isolated using mirVana™ miRNA Isolation Kit (Thermo Scientific) with Acid-Phenol:Chloroform (Thermo Scientific), following the manufacturer’s protocol. After the quality of miRNA was examined via bio-analysis, small RNA library construction and deep sequencing were performed at BGI (Shenzhen, Guangdong, China). Briefly, after PAGE purification, small RNA molecules between 16 and 30 bases were collected and ligated with a pair of Solexa adaptors to their 5′ and 3′ ends respectively. The small RNAs were then amplified using the adaptor primers for 17 cycles. Amplicons of around 90 bp (small RNA + adaptors) were isolated from the agarose gel and were used directly for cluster generation and sequencing analyses using Illumina Hiseq 2000 according to the manufacturer’s instructions.

### MiRNA arm-switching detection and differential miRNA expression

FastQC was run as quality control [56] for the 72 small RNA datasets. Adaptor sequences were trimmed from small RNA sequencing reads, and reads with the Phred quality score less than 20 were removed. The expression levels of the 5p and 3p arms of soybean miRNAs from miRBase (Release 22.1) were calculated based on the number of sequencing reads mapped to the respective arm region in the miRNA hairpin using bowtie/miRDeep2 [57, 58]. The miRNAs having either arm with absolute counts > 50 were included in the arm-switching analysis. The formula, ω = 5p/(5p + 3p), where 5p and 3p refer to the number of predicted 5p arm and 3p arm, respectively, was adopted to calculate the arm selection value, ω, which ranged from 0 to 1. Smaller ω values indicate higher tendencies of 3p preference and larger values indicate higher tendencies of 5p preference. We adopted ω < 0.3 as the indicator of 3p dominance and ω > 0.7 as the indicator of 5p dominance. The shift of arm dominance is defined as arm switching [24]. The expression raw read table, calculated as previously described, was submitted to Degust [59] for the comparative analyses of differential miRNA gene expression using the edgeR method [60]. (Degust link: https://degust.erc.monash.edu/degust/compare.html?code=708756e3e3e0ad71f68186a9e83f19c2#/.)

### Interaction prediction between miRNAs and long noncoding RNAs (lncRNAs)

The soybean lncRNA annotation was retrieved from a previously published soybean lncRNA catalog [44]. The published time-series transcriptome data upon salt treatment [43, 45] were processed as described [44]. Briefly, adapter and quality trimming were performed using Trimmomatic 0.36 [61]. Then, the clean reads of C08 and W05 were mapped to the reference genomes of Williams 82 (*G. max*) [62] and W05 (*G. soja*) [45], respectively, using TopHat v2.1.1 [63]. With the read mapping results, Cufflinks [64, 65] was used to perform annotation-free transcriptome assembly. The assembled transcripts were merged using the Cufflinks script, “cuffmerge”, to produce unified transcript sets separately in both genomes. These transcripts were then compared against the protein-coding transcripts, as well as small non-coding transcripts as predicted by RNAmmer v1.2 [66], tRNAscan-SE v2.0 [67] and Infernal v1.1.2 [68] with Rfam v13.0 database [69], and searched against the nonredundant protein database from NCBI using BLASTx [70] to identify potential lncRNAs. Trinity [71] was used for de novo transcriptome assembly with the clean reads, separately for the C08 and W05 data. All Trinity-assembled transcripts were aligned to the two reference genomes using GMAP v2018-03-25 [72]. The above-mentioned potential lncRNAs were used for downstream analyses only when they were supported by both the Trinity-assembled transcripts and the lncRNA catalog. The differential gene expression analysis was performed using the Cufflinks script, “cuffdiff”, with the criteria set at |log_2_FC| > 1 and *q*-value < 0.05. Target prediction between miRNA and lncRNA was performed by RNAhybrid [73], based on the seed region pair-matching with default parameters, and by LncMirNet [74] based on deep-learning of ribonucleic acid sequences, respectively. For the comparison across the time-series of salinity treatment, parameters were set to screen for differentially expressed miRNAs and lncRNAs to investigate potential interactions between them. For lncRNAs, |log_2_FC| ≥ 1 and q-value ≤0.05 were used as screening criteria for differential expression. For miRNAs, counts per million (CPM) ≥ 50 in at least one sample, |log_2_FC| ≥ 1, and false discovery rate (FDR) cut-off ≤0.05 are the criteria used for differential expression screening.

### Target gene prediction of miRNAs

Target predictions between miRNAs and the 5′ untranslated regions (UTR), coding sequence (CDS) and 3’UTR of protein-coding genes were performed by psRNATarget [75]. The functional term annotations were performed using eggNOG [76] with default parameters and taxon restricted to Fabids (Taxon ID:91835). Genes were assigned with Gene Ontology (GO), EuKaryotic Orthologous Groups (KOG), Kyoto Encyclopedia of Genes and Genomes (KEGG), and KEGG Orthology (KO) terms. Functional enrich of mirna target gene was tested using function ‘compareCluster()’ in R package ‘clusterProfiler’ v.3.16.1 [77] under the environment of R 4.0.4 [78]. Significantly enriched terms were determined with pvalueCutoff = 0.05, pAdjustMethod = “BH”, and qvalueCutoff = 0.2. Data was visualised using R packages ‘ggplot2’ [79].

### Labeled miRNA pull-down (LAMP) assay

The predicted interactions between miRNAs and lncRNAs were validated by biotin-labeled miRNA pull-down (LAMP) assay [80] using Dynabeads®M-280 Streptavidin (Invitrogen™) and biotin-labeled gma-miR166i-5p and gma-miR394a-3p (Integrated DNA Technologies [IDT]), followed by RT-PCR. The primers of four target lncRNAs for PCR are listed in Supplementary Table 1. RNA from the leaf of C08 under salt treatment for 24 h was the starting material and the RNA pulled-down products were reverse-transcribed using the iScript cDNA Synthesis Kit (BIO-RAD) following the manufacturer’s protocol to obtain the cDNAs. Those originating from the four target lncRNAs were amplified by PCR and separated on agarose gel. The target bands were excised and purified by QIAquick gel extraction kit (Qiagen) following manufacturer’s protocol and sent for Sanger sequencing for confirmation.

### Dual luciferase reporter (DLR) assay

Genomic DNA and cDNA were used to amplify microRNA hairpins with flanking sequences, target genes and lncRNAs. Primers information were listed in Supplementary Table 1. Sequences used in DLR assay were listed in Supplementary Table 7. MicroRNAs were cloned into pAC5.1 vector, while the target genes and lncRNAs were cloned into psicheck-2 vector. Dual luciferase reporter assay was conducted in *Drosophila* S2 cells as previously described [81] with following modifications: *Drosophila* S2 cells were cultured in Shields and Sang M3 Insect Medium (Sigma) supplemented with 10% (v/v) heat-inactivated fetal bovine serum (Gibco, Life Technologies) and 1: 100 penicillin-streptomycin (Gibco, Life Technologies). Measurement was performed at 36-48 h post-transfection by Tecan Spark 10 M Microplate Reader (Eastwin International Trading Ltd) with two technical replicates four biological replicates.

## Supplementary Information

**Additional file 1: Supplementary Figure 1.** Mapped reads of miR166m in individual samples. **Supplementary Figure 2.1.** PHRED Score Distribution. **Supplementary Figure 2.2.** Read Length Distribution. **Supplementary Figure 2.3.** Quality Control Statistics. **Supplementary Figure 2.4.** RNA Type. **Supplementary Figure 2.5.** miRNA Complexity. **Supplementary Figure 2.6.** Contamination. **Supplementary Figure 3.** Dual luciferase reporter assay.

**Additional file 2: Supplementary Table 1.** Oligos and primer sequences used in this study. **Supplementary Table 2.** Number of raw reads generated in the datasets in this study. **Supplementary Table 3.** Differential miRNA expression in different samples as shown in Fig. 3. **Supplementary Table 4.** Differential lncRNA expression in different samples as shown in Fig. 5B and C. **Supplementary Table 5.** Target predictions of miR166m-5p and miR166m-3p in C08 and W05. **Supplementary Table 6.** Genes selected for validation. **Supplementary Table 7.** Sequences used in the DLR assay.

## Data Availability

The raw reads of the 72 small RNA samples generated in this study have been deposited to the NCBI database under the BioProject accessions: PRJNA720229.
