# Supplementary material for: Differential microRNA expression, microRNA arm switching, and microRNA:long noncoding RNA interaction in response to salinity stress in soybean
Source: BMC Genomics. 2022 Jan 20;23:65. doi: 10.1186/s12864-022-08308-y (PMC8780314; doi:10.1186/s12864-022-08308-y)
Supplement: Supplementary file 1 — Additional file 1: Supplementary Figure 1. Mapped reads of miR166m in individual samples. Supplementary Figure 2.1. PHRED Score Distribution. Supplementary Figure 2.2. Read Length Distribution. Supplementary Figure 2.3. Quality Control Statistics. Supplementary Figure 2.4. RNA Type. Supplementary Figure 2.5. miRNA Complexity. Supplementary Figure 2.6. Contamination. Supplementary Figure 3. Dual luciferase reporter assay. [file 12864_2022_8308_MOESM1_ESM.docx]

**Supplementary Figure 1.** Mapped reads of miR-166m in individual samples.


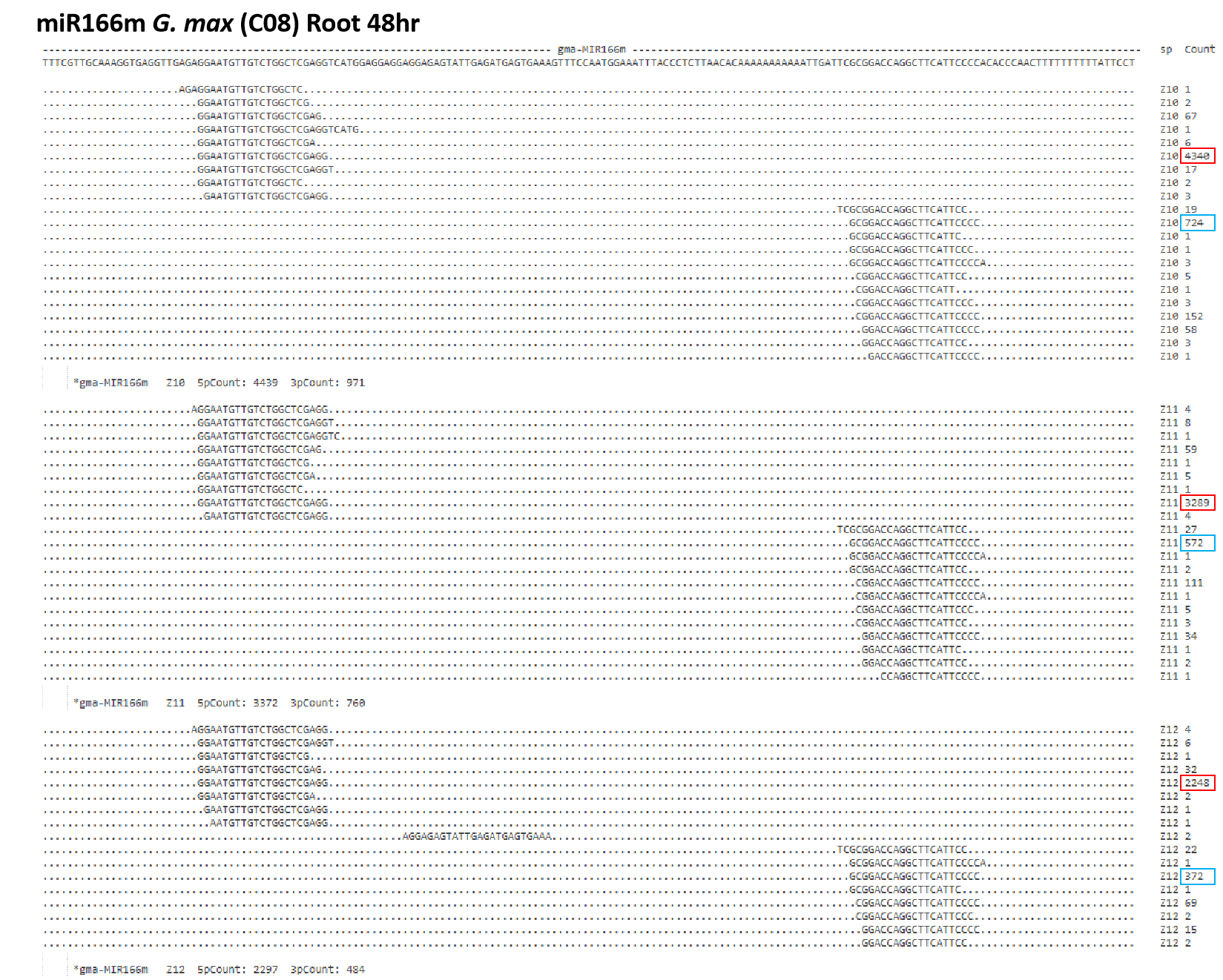


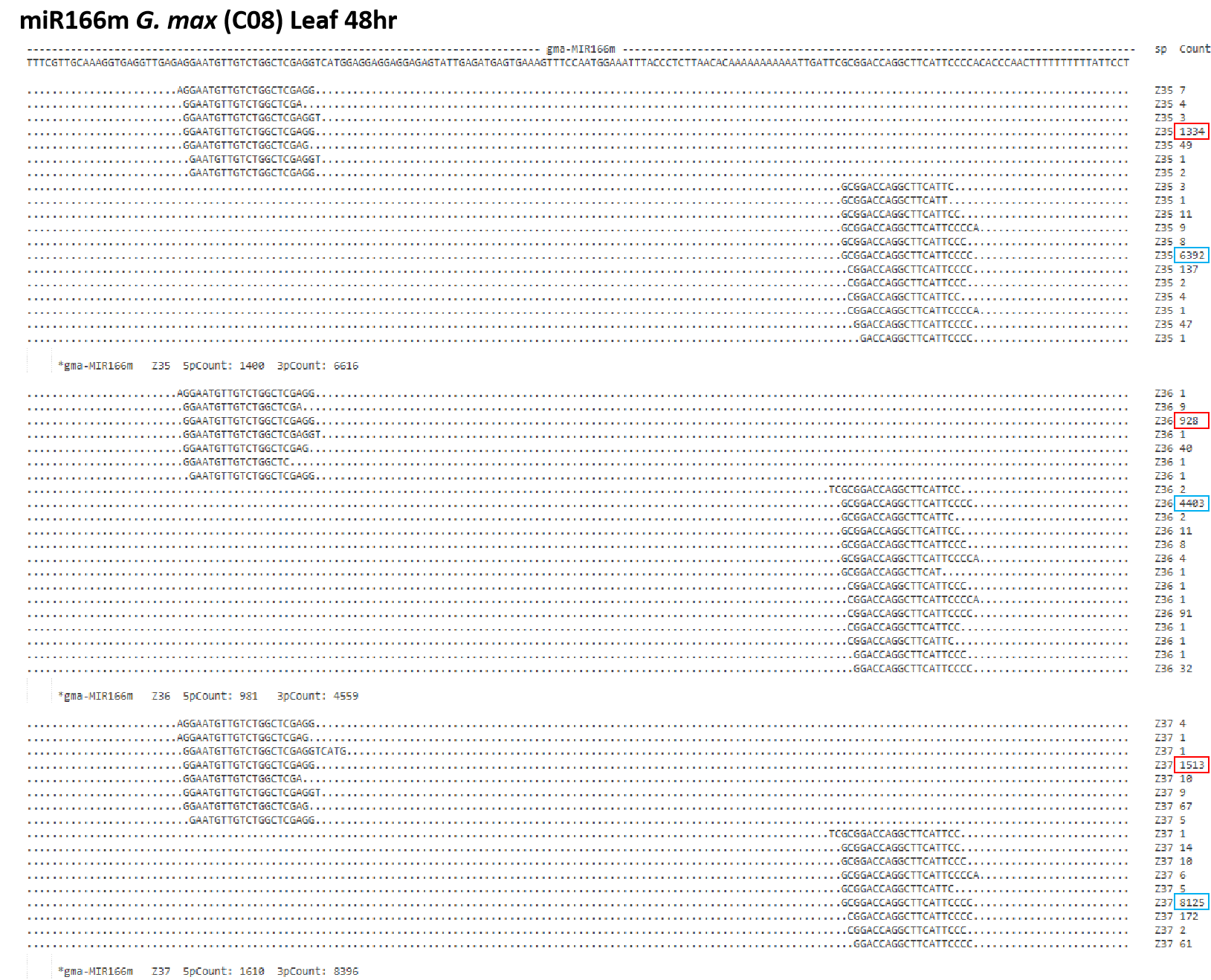


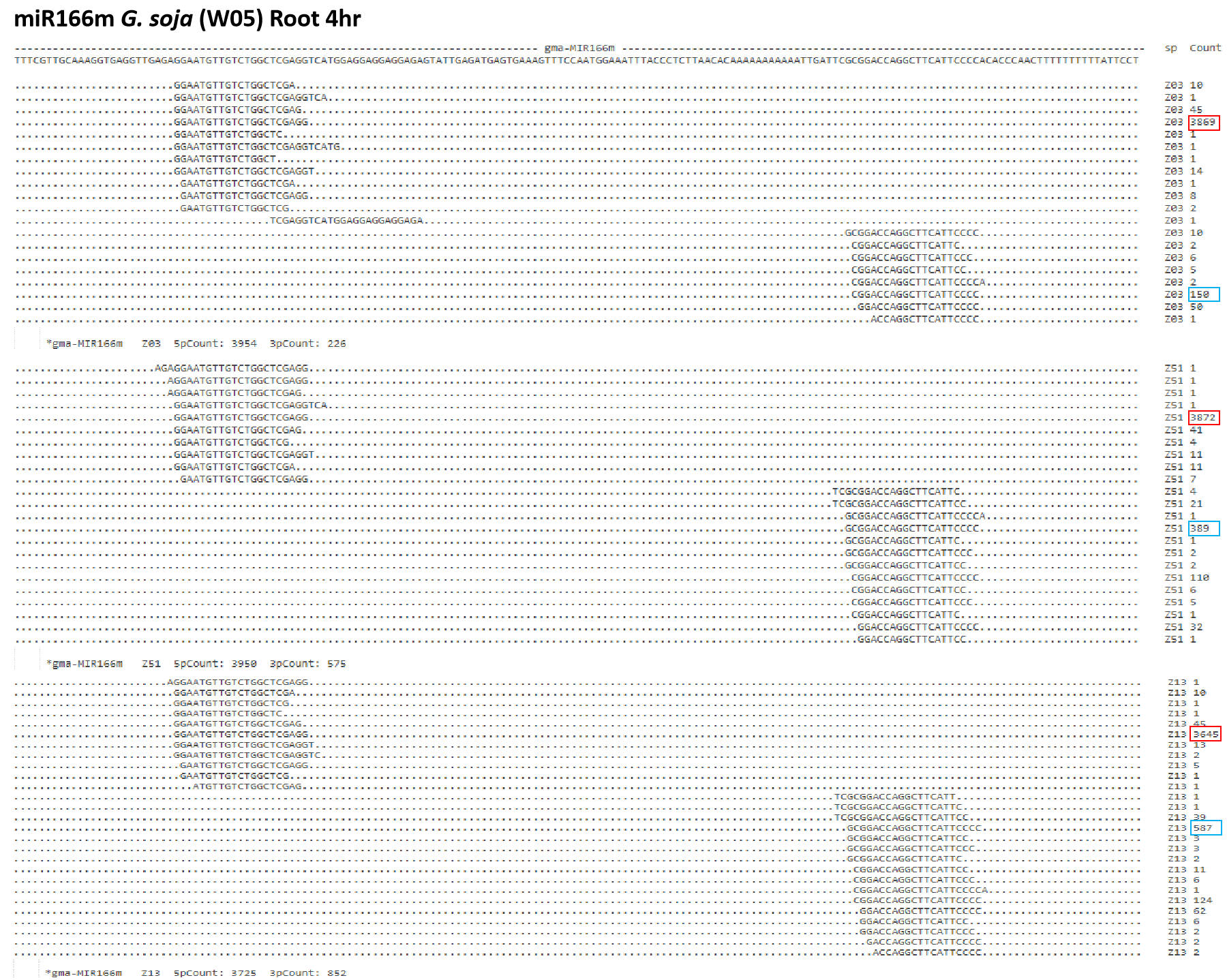


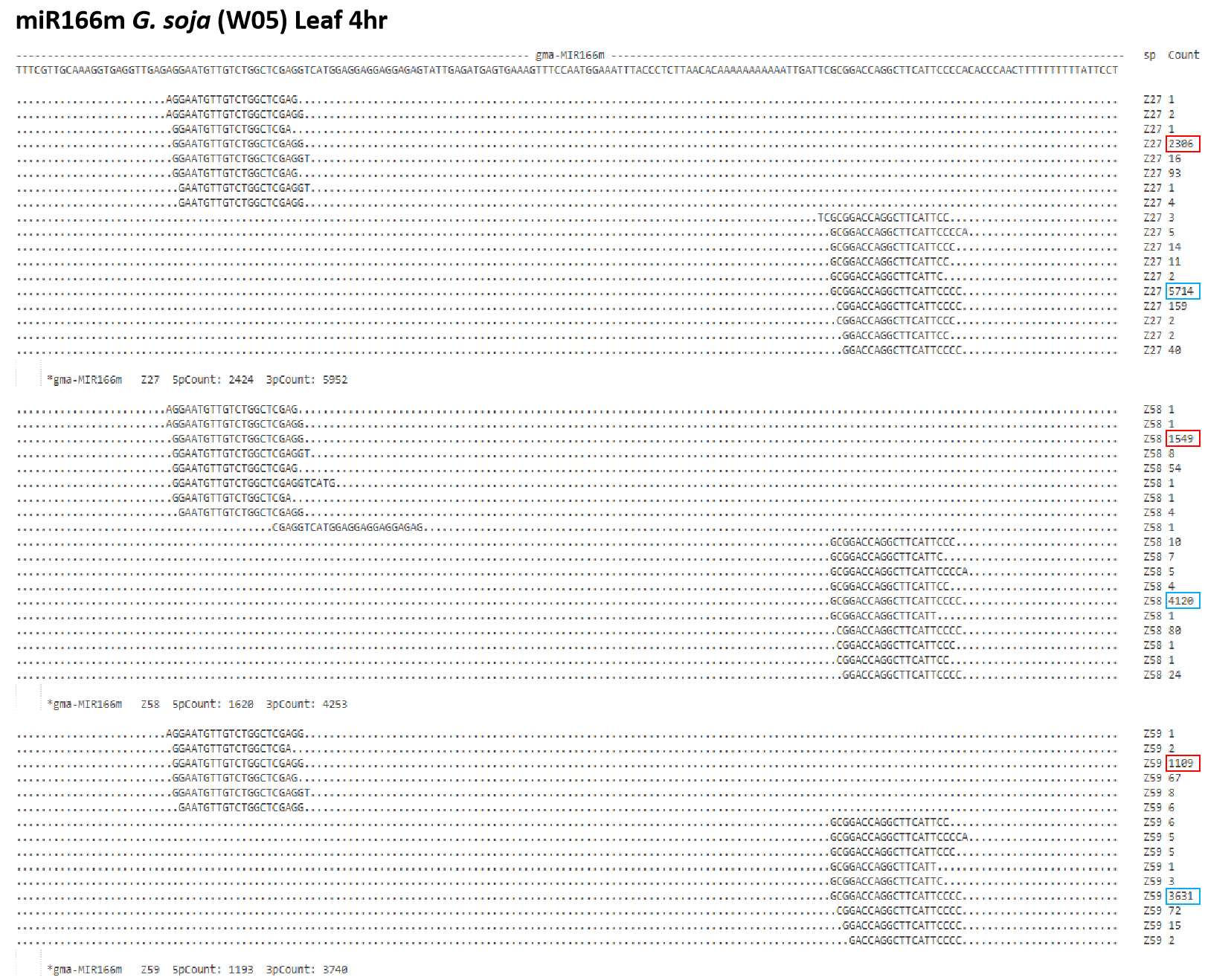


**Supplementary Figure 2.1.** PHRED Score Distribution.


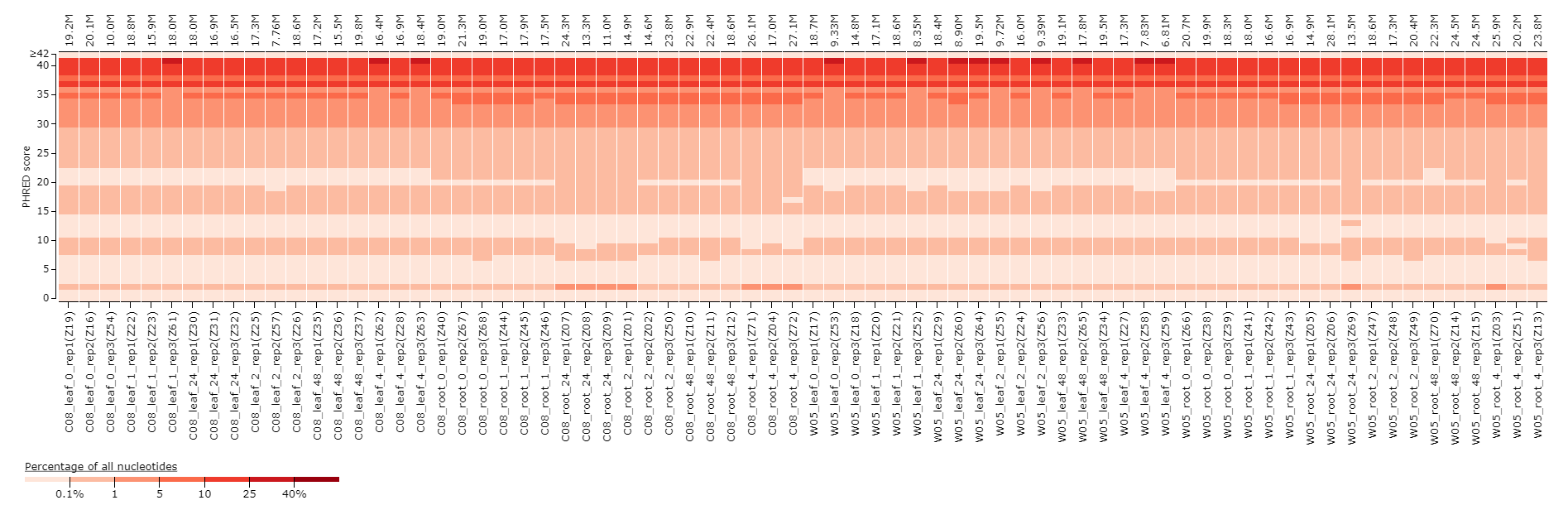


**Supplementary Figure 2.2.** Read Length Distribution.


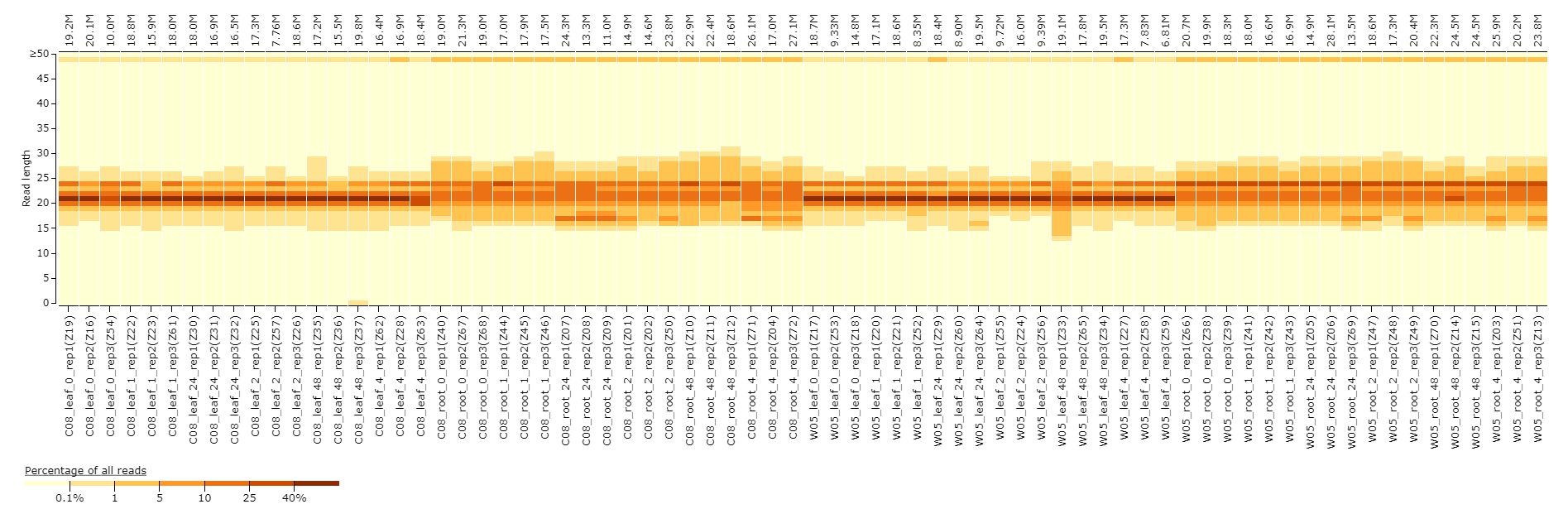


**Supplementary Figure 2.3.** Quality Control Statistics.


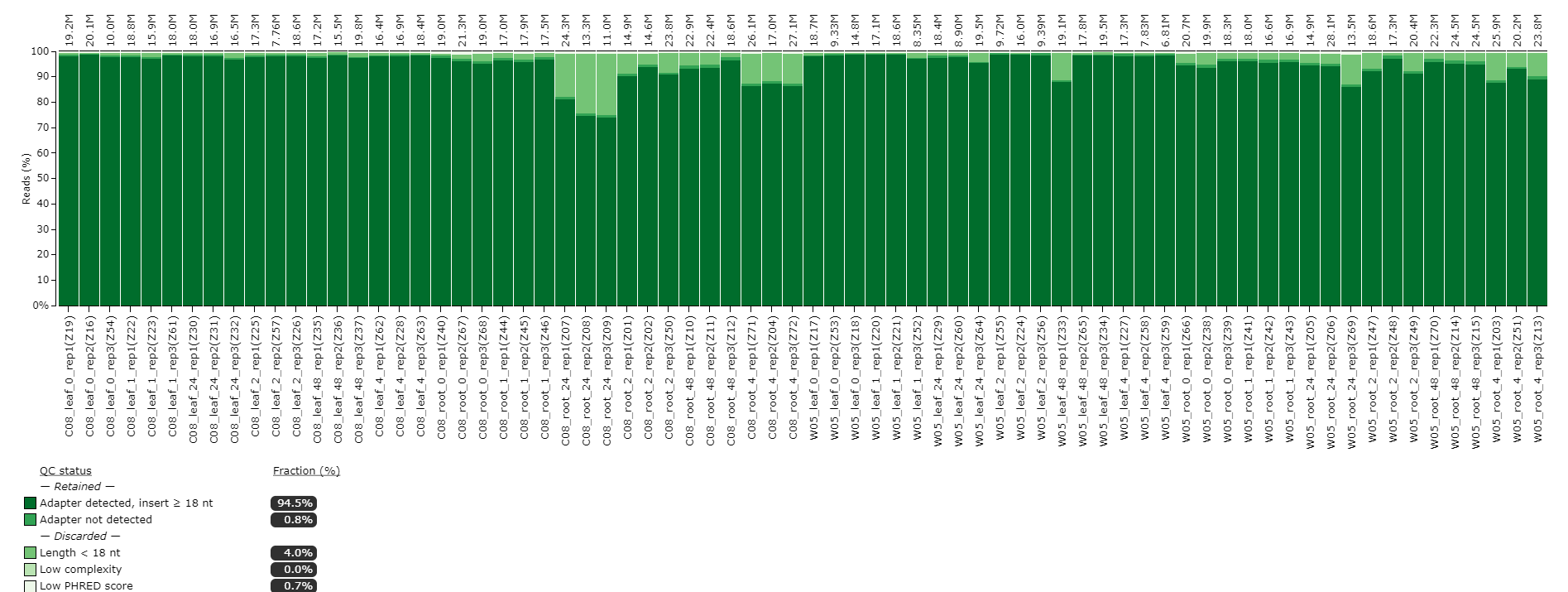


**Supplementary Figure 2.4.** RNA Type.


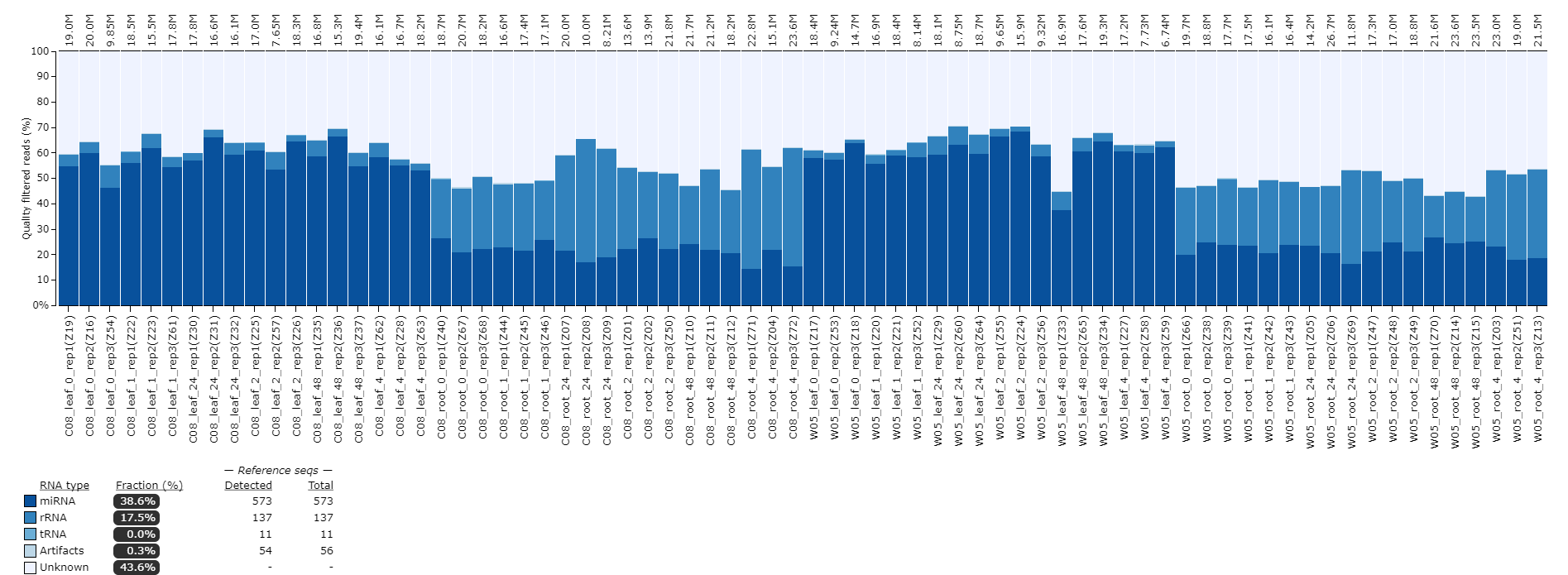


**Supplementary Figure 2.5.** miRNA Complexity.


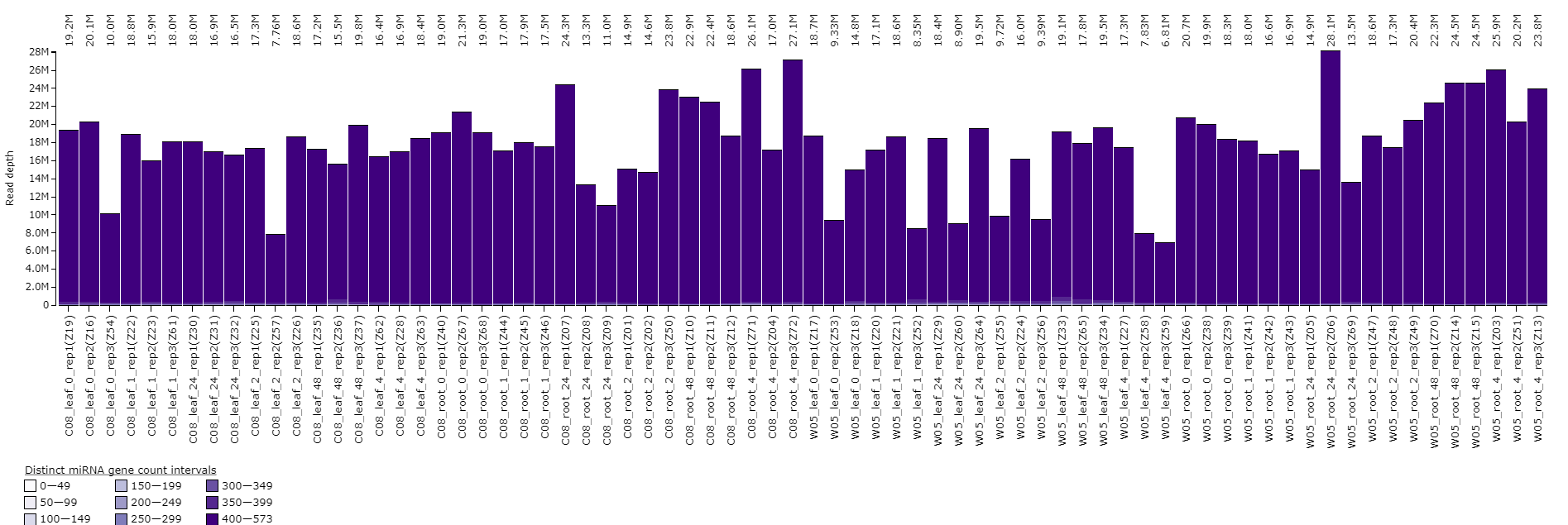


**Supplementary Figure 2.6.** Contamination.


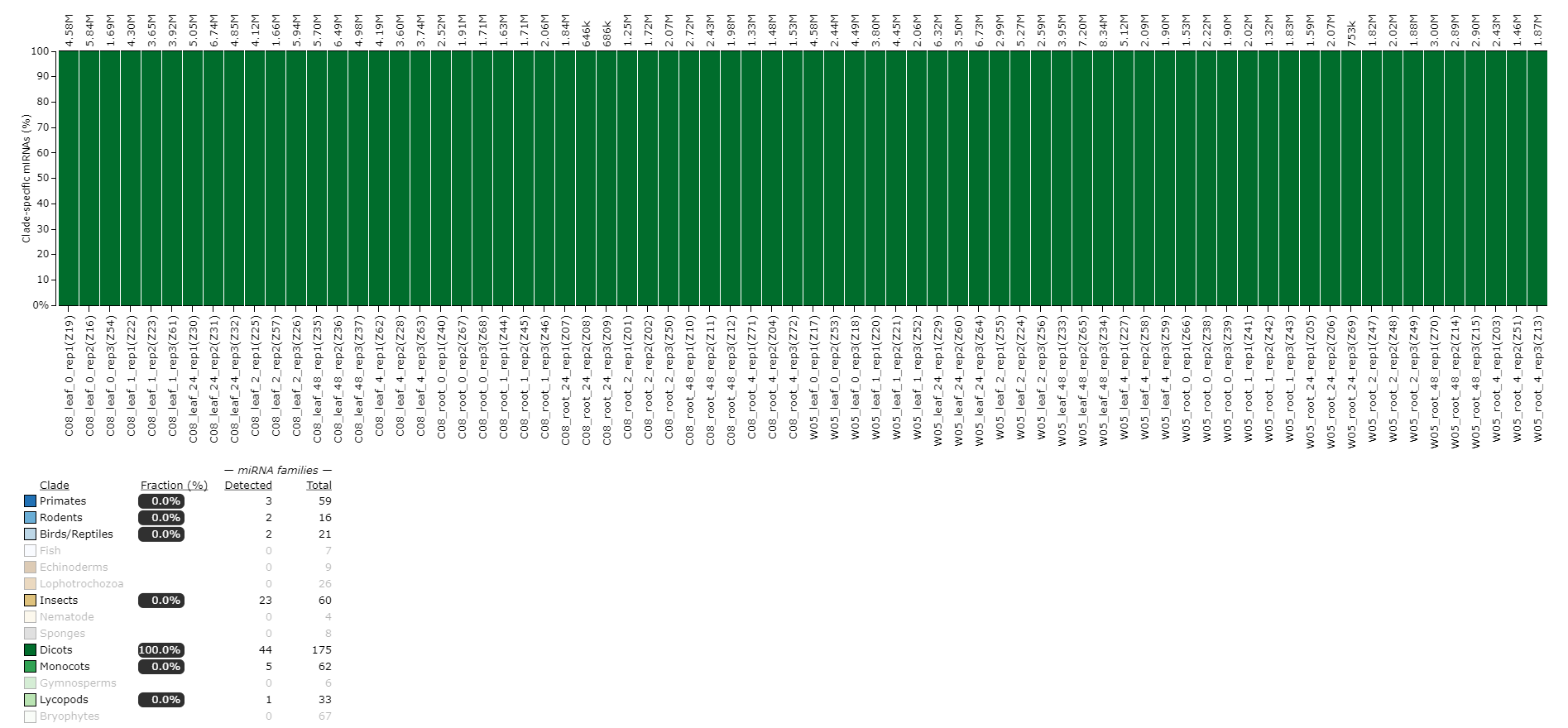


**Supplementary Figure 3.** Dual luciferase reporter assay
